# Effect of Dynamical Motion in *ab Initio* Calculations of Solid-State Nuclear Magnetic and Nuclear Quadrupole Resonance Spectra

**DOI:** 10.1021/acs.chemmater.4c00883

**Published:** 2024-07-19

**Authors:** Kamal Wagle, Daniel A. Rehn, Ann E. Mattsson, Harris E. Mason, Michael W. Malone

**Affiliations:** † Computational Physics Division, Los Alamos National Laboratory, Los Alamos, New Mexico 87545, United States; ‡ Chemistry Division, Los Alamos National Laboratory, Los Alamos, New Mexico 87545, United States; ¶ Materials Physics and Applications Division, Los Alamos National Laboratory, Los Alamos, New Mexico 87545, United States; ∥ Center for Nonlinear Studies, Los Alamos National Laboratory, Los Alamos, New Mexico 87545, United States

## Abstract

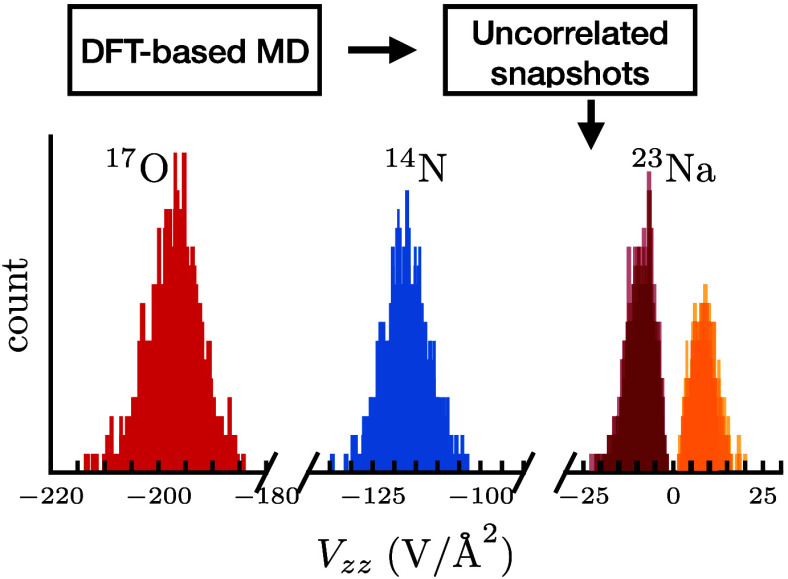

Solid-state nuclear magnetic resonance (SSNMR) and nuclear quadrupole resonance (NQR) spectra provide detailed information about the electronic and atomic structure of solids. Modern *ab initio* methods such as density functional theory (DFT) can be used to calculate NMR and NQR spectra from first-principles, providing a meaningful avenue to connect theory and experiment. Prediction of SSNMR and NQR spectra from DFT relies on accurate calculation of the electric field gradient (EFG) tensor associated with the potential of electrons at the nuclear centers. While static calculations of EFGs are commonly seen in the literature, the effects of dynamical motion of atoms in molecules and solids have been less explored. In this study, we develop a method to calculate EFGs of solids while taking into account the dynamics of atoms through DFT-based molecular dynamics simulations. The method we develop is general, in the sense that it can be applied to any material at any desired temperature and pressure. Here, we focus on application of the method to NaNO_2_ and study in detail the EFGs of ^14^N, ^17^O, and ^23^Na. We find in the cases of ^14^N and ^17^O that the dynamical motion of the atoms can be used to calculate mean EFGs that are in closer agreement with experiments than those of static calculations. For ^23^Na, we find a complex behavior of the EFGs when atomic motion is incorporated that is not at all captured in static calculations. In particular, we find a distribution of EFGs that is influenced strongly by the local (changing) bond environment, with a pattern that reflects the coordination structure of ^23^Na. We expect the methodology developed here to provide a path forward for understanding materials in which static EFG calculations do not align with experiments.

## 1 Introduction

Solid-state nuclear magnetic resonance (SSNMR)^1−5^ and nuclear quadrupole resonance (NQR)^6^ spectroscopies are powerful analytical chemistry methods that provide detailed information about the local electronic structure of solid materials. SSNMR and NQR share the ability to probe the electric field gradients (EFGs) of electrons at the atomic nucleus. Fundamental information about the electronic structure of molecules and solids can be obtained from such studies.^7−9^ SSNMR and NQR can also be used to provide detailed structural information^10^ which can allow phase identification and differentiation among chemically similar materials such as structural polymorphs.^11−13^ These methods can provide chemical fingerprints that can be useful in a range of applications such as nondestructive characterization of pharmaceuticals^14,15^ as well as detecting illicit narcotics^16,17^ and explosive substances.^18,19^

SSNMR and NQR can both probe the interaction between EFGs and quadrupolar nuclei (i.e., nuclei with spin *I* ≥ 1). Both spectroscopies operate in the radiofrequency (RF) region of the electromagnetic spectrum and principally use resonant RF pulses to induce AC magnetic fields from the target nuclei that are then detected with a magnetometer. In SSNMR, the dominant mechanism is the Zeeman Hamiltonian: the interaction of a nuclear magnetic moment with an applied magnetic field. The quadrupole Hamiltonian, *H*_*Q*_, defined below, acts as a perturbation. In practice, spectra are collected at the highest magnetic field available to the experimentalist. This typically requires large, cryogenically cooled superconducting magnets. Further, the spectra are convoluted with contributions from chemical shift, and nuclear dipole coupling which can potentially distort the spectra. In NQR, the dominant mechanism is the quadrupole Hamiltonian: the interaction of the nuclear charge distribution with the local electric field gradient. Since NQR directly probes the quadrupole interaction, it provides a more precise measurement of EFGs than SSNMR. However, since the quadrupole interaction is only a perturbation in SSNMR, the spectra of quadrupolar nuclei are more easily, if comparatively roughly, observed via SSNMR. The challenge for observing NQR signals of new materials is that some knowledge of the EFGs is necessary before attempting to interrogate a sample. This information is not known *a-priori*.

A number of studies have investigated the effect of ionic motion on EFGs. One approach that has been explored previously is the use of path integral molecular dynamics (PIMD).^20,21^ PIMD includes quantum nuclear effects, which can be especially important for light elements, and has been used to calculate NMR parameters. Additionally, quantum nuclear effects can be included through stochastic sampling.^22−24^ However, these methods are computationally intensive, and are not expected to be as important for NaNO_2_ as they would be for systems containing lighter elements, including hydrogen. In addition, machine learning (ML) based potentials have been used in the context of NMR calculations^25,26^ due to their low computational cost and applicability to larger systems. However, we choose to use DFT-MD for this study in order to avoid the possibility of additional uncertainties associated with ML potentials from entering into our analysis.

Density functional theory (DFT) is frequently used to calculate the quadrupole interaction for SSNMR and NQR spectra.^27−31^ Blaha et al.^27^ first implemented the DFT method to calculate EFGs in the (all electron) full potential linear augmented plane wave (FP-LAPW) method. The methodology to calculate EFGs using the projector augmented wave (PAW)^32−34^ method was later developed, which has the benefit of allowing for faster calculations while still maintaining an accuracy comparable to all-electron calculations of EFGs. Since then, the PAW approach has been used extensively, with some examples found in refs. (28 and 35−37).

A disconnect between the physical reality of solid materials and DFT-based EFG calculations still exists in much of the literature. A typical study will use DFT at 0 K to first perform a structural relaxation starting from an experimentally determined crystal structure. This final structure can then be used in a static EFG calculation and can be thought of as a static snapshot of the chemical system. The use of static DFT to predict EFGs suffers from limitations if atoms or molecules in the studied structure experience significant dynamics.^38^ Additionally, atoms vibrate at THz frequencies, whereas SSNMR and NQR probe interactions on kHz to MHz frequencies. Therefore, the spectra produced are not a single snapshot, but rather the time average of these THz fluctuations. Several studies^39−46^ have started to recognize this disconnect and include atomic motion in the calculation of SSNMR and NQR. In this study we perform a more detailed microscopic analysis of how EFG eigenvalues and eigenvectors fluctuate at finite temperature and present a real-space picture of how these fluctuations occur, as well as how this may help guide experimental studies.

In this work, we use DFT-based molecular dynamics (DFT-MD) to simulate the dynamical motion of atoms in sodium nitrite, NaNO_2_. This method calculates statistically averaged EFGs through uncorrelated snapshots of atomic configurations. NaNO_2_ is one of the standard materials to test NQR/SSNMR signals. We also choose NaNO_2_ because each species (Na, N, and O) has well-known experimental spectra and data from other DFT calculations that have been compared with experiments. Here, we find that our method to include the dynamical motion of the atoms allows us to calculate EFGs in ^14^N, ^17^O, and ^23^Na in better agreement with experiment, compared to static calculations. Additionally, the method we present captures the complex behavior of EFGs in ^23^Na due to its changing bonding environment, an effect that is completely absent in static calculations. We expect the method we develop to shed light on the EFGs of more complicated quantum systems where static calculations fail.

## 2 Methodology

### 2.1 EFG and SSNMR/NQR: Theory

The interaction between a nuclear charge ρ_*n*_(**r**) and the electrostatic potential *V*(**r**) of the surrounding electrons is given by the Hamiltonian,
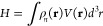
1By performing a series expansion of the potential *V*(**r**) the remaining, second order terms provide the quadrupolar Hamiltonian:^5,47,48^
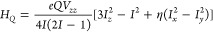
2Here *Q* is the nuclear quadrupole moment, *e* is the electronic charge, *I* is the nuclear spin, and both η and the coordinate system for the angular momentum operators *I*_*x*_, *I*_*y*_, and *I*_*z*_ are defined below.

The expansion of *V* (*r*) evaluated at the position of the nucleus (**r** = 0) results in a tensor,
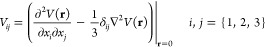
3This tensor is symmetric and traceless. The latter constraints means that only two terms are necessary to define the tensor. We work in a coordinate system that diagonalizes the tensor such that
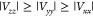
4Note that the ordering of |*V*_*xx*_| and |*V*_*yy*_| is sometimes found in the reverse order to our convention. *V*_*zz*_ gives a measurement of the deviation of the electron density near the nucleus from spherical symmetry. It is sometimes expressed via the quadrupole coupling constant, *C*_*Q*_ = *eQV*_*zz*_/*h*, where *h* is Planck’s constant, for ease in comparison with experiments. The second relevant term for defining the EFG tensor is the asymmetry parameter, η, of the diagonalized EFG, defined as
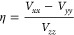
5η gives information about the point group of the site under consideration. If the site under consideration has cubic, tetragonal, or hexagonal symmetry, η is zero. In other words, η is zero if there is at least a 3-fold rotational symmetry in the proximity of the nucleus.

### 2.2 Computational Methods

Our computational approach makes use of DFT-MD to study SSNMR/NQR signals in NaNO_2_. To obtain EFGs, we take uncorrelated snapshots from the MD trajectory (trajectory meaning a sequence of atomic configurations) and calculate EFGs of individual atoms in NaNO_2_ from these snapshots. With this approach we obtain statistically meaningful information about the effect of atomic motion on the EFGs in NaNO_2_.

All calculations are performed using VASP version 5.4.4^49−52^ using the projector augmented wave (PAW)^32−34^ method and PBE^53^ for the exchange-correlation functional. In addition, we use the so-called *GW* PAW potentials in VASP, which are generally of higher quality and are designed for calculating scattering properties at higher energies in *GW*(54) calculations. These PAW potentials treat the valence configurations as 2*s*^2^2*p*^3^ for nitrogen, 2*s*^2^2*p*^4^ for oxygen, and 2*s*^2^2*p*^6^3*p*^1^ for sodium.

**Figure 1:**
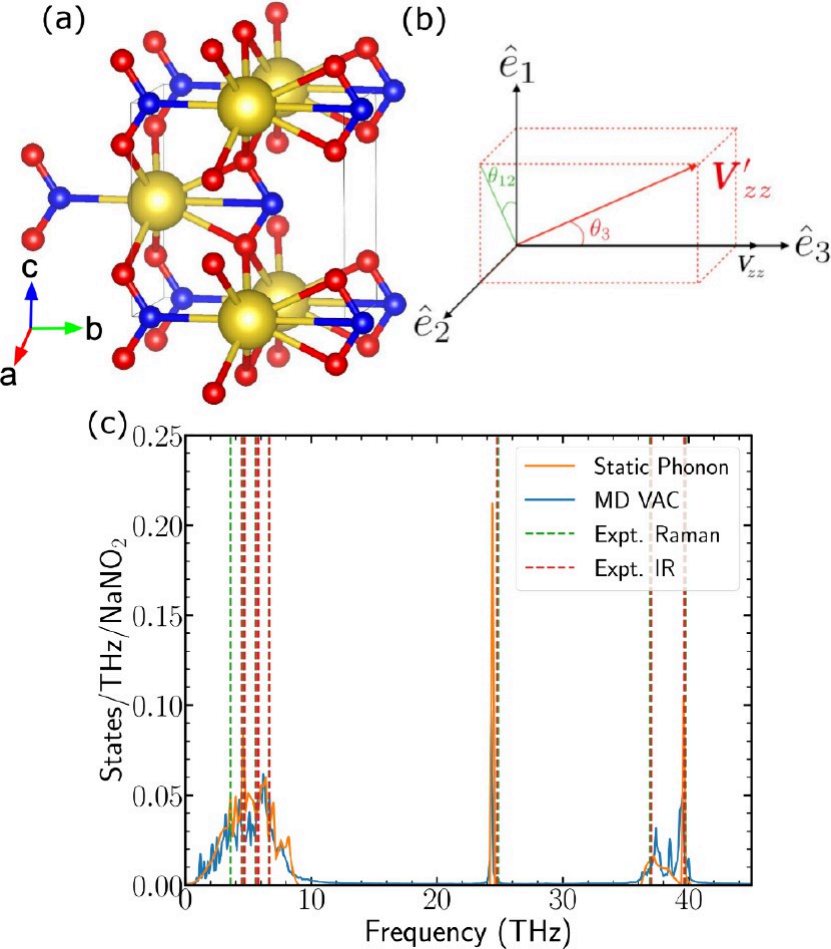
(a) The unit cell of NaNO_2_ with nitrogen in blue, oxygen in red, and sodium in yellow. (b) Schematic of the direction of dynamic ***V***_*zz*_*′* and static ***V***_*zz*_, angle θ_3_ between ***V***_*zz*_*′* and ***V***_*zz*_, and angle θ_12_ in the plane perpendicular to static ***V***_*zz*_. Normalized eigenvectors in the static frame are labeled *ê*_*i*_ here. (c) Comparison of our calculated Phonon density of states in NaNO_2_ obtained from velocity autocorrelation (VAC) and static phonon calculation with experimentally measured IR and Raman spectra obtained from refs. (58−60).

The experimental NaNO_2_ structure we use is taken from ref. (55) which can be found in the inorganic crystal structure database (ICSD),^56^ shown in Figure 1(a). This orthorhombic structure (space group #44, *Im2m*)^57^ is stable up to 436 K, well above 300 K used in DFT-MD simulations.

Using the experimental (8 atom, 2 formula units of NaNO_2_) structure, we first performed a relaxation to find the PBE-predicted zero temperature lattice constants. These relaxations were performed using a plane wave energy cutoff *E*_cut_ = 450 eV, a convergence criteria for the energy difference between successive electronic steps *E*_diff_ = 10^–5^ eV, and the k-point mesh was set to 12 × 12 × 12 using a Γ-centered Monkhorst–Pack^61^ mesh. Relaxations were restarted 3 times to account for basis set changes upon relaxation and ensure a fully converged relaxed structure. After these relaxations, we performed a static calculation with tighter convergence criteria to calculate the static EFG tensors. For these static calculations we used *E*_cut_ = 600 eV, *E*_diff_ = 10^–7^ eV, and the k-point mesh was set to 16 × 16 × 16. The calculated EFG eigenvalues are shown in Table 1. Note that these calculations are in close agreement with results presented by Ansari et al. in ref. (62) which found that for nitrogen, *V*_*zz*_ calculated using different PAW potentials falls within the range 117–120 V/Å^2^. The EFGs we calculate after relaxing the structure are also in close agreement with EFGs calculated using fixed, experimentally determined room temperature structures in refs. (63−70) available in the ICSD database.^56^ We chose to use the relaxed structures in order to minimize any internal stresses in calculations.

**Table 1:** Comparison of Calculated Static and DFT-MD Values of *V*_*zz*_ for Nitrogen, Oxygen, and Sodium with the Experimental^78−82^ Results^a^

	Static (V/Å^2^)	DFT-MD (V/Å^2^)	Expt. (V/Å^2^)
N	118.3	115.2	111.0^78^
O	196.3	193.0	178.7^82^
Na	6.1	(see Sec. 3.3)	4.4^80,81^

a See Sec. 3.3 for the DFT-MD result for Na.

The DFT-MD simulations performed are used to generate an MD trajectory that can subsequently be sampled for calculation of EFGs. Because one goal of our study is to compare the static and dynamic EFGs, we use the same lattice constants for both the static and dynamic calculations; in other words, we neglect the role of thermal expansion in our study in order to make a cleaner comparison of static and dynamic EFGs. Nonetheless, the methodology we present can be used to study the role of thermal expansion on EFGs; it would simply require applying the method we present at a series of volumes *V* and temperatures *T* along the 1 atm isobar.

In order to ensure highly accurate EFGs, we do not calculate EFGs “on the fly” during the DFT-MD simulations. There are two reasons for this: 1) the EFGs require much tighter convergence criteria than what is needed to generate the MD trajectory and 2) adjacent MD steps are highly correlated with each other, so that calculating EFGs at every step is neither necessary nor helpful from a statistical point of view. Because of these issues, we instead take an approach where we run DFT-MD simulations to generate an MD trajectory and then calculate EFGs from uncorrelated snapshots taken from the MD trajectory. Addressing the problem in this way necessitates studying two different convergence properties: A) convergence of the vibrational properties within the MD trajectory to ensure that the phonons of the system are sampled accurately and reflect the vibrational properties of the real physical system and B) convergence of the EFG calculations taken from individual snapshots. We discuss each of these below.

To ensure the vibrational properties from our MD trajectory are sufficiently accurate, we calculate the phonon density of states (PDOS) for DFT-MD simulations of different cell sizes and k-mesh sizes. The PDOS is calculated using the velocity autocorrelation (VAC) of the MD trajectories, in which the power spectrum of the VAC gives the PDOS.^71^ The calculated PDOS from DFT-MD simulations can be compared to static phonon calculations to validate that the DFT-MD and phonon calculations give approximately the same PDOS, and these each can be approximately verified to represent reality by comparing the PDOS with experimental infrared (IR) and Raman spectra, also shown in Figure 1. In studying this, we performed DFT-MD simulations for both 64 atom (2 × 2 × 2) and 216 atom (3 × 3 × 3) supercells using two different k-mesh sizes: Γ-point only and a 2 × 2 × 2 k-mesh. We found that the PDOS of the 216 atom cells for both the Γ point and 2 × 2 × 2 k-mesh sizes were approximately equal, and we decided to use the 216 atom cell with a 2 × 2 × 2 k-mesh for all DFT-MD calculations in this study. The PDOS we calculated was also in close agreement with a static phonon calculation using finite displacements as implemented in the Phonopy^72,73^ software, using a 216 atom cell. Figure 1(c) shows close agreement between the phonon frequencies obtained using both DFT-MD and static phonon calculations, and that these results are in close agreement with experimentally measured IR and Raman spectra from refs. (58−60).

The DFT-MD simulations use an *NVT* ensemble with a Nosé mass thermostat to control the temperature fluctuations during the simulation.^74−76^ In our calculations, we chose a Nosé mass corresponding to a period of 40 time steps. This means that at every 40th time step, the system is recoupled with the heat bath, which helps to control temperature fluctuations. The time step was set to 1 fs, *E*_diff_ was set to 10^–4^ eV, and *E*_cut_ was set to 450 eV. The DFT-MD simulation was run for ∼10 ps (10,400 total timesteps) after an initial equilibration period, at a fixed temperature of 300 K. The total cost of this approach is around 150,000 CPU-hours.

After generating the DFT-MD trajectory, we calculated EFG tensors from snapshots in 100 fs intervals. The 100 fs intervals were found to be uncorrelated by analyzing the standard error in the energy using a block averaging procedure (details of the approach are discussed in ref. (77)). A total of 104 snapshots were used to calculate the EFGs, each snapshot consisting of 216 atoms: 54 Na atoms, 54 N atoms, and 108 O atoms. These EFG calculations are performed using *E*_cut_ = 500 eV, *E*_diff_ = 10^–7^ eV, and k-point mesh of size 4 × 4 × 4.

We study the effect of the dynamical motion of atoms on the EFG parameters by investigating how the eigenvalues and eigenvectors of the EFG tensor change throughout the simulation. We define the eigenvalues of the static structure as *V*_*ii*_ (*i* = 1, 2, 3 = *x*, *y*, *z*). The normalized eigenvectors associated with *V*_*ii*_ are labeled *ê*_*i*_. This allows us to use a compact notation ***V***_*ii*_ = *V*_*ii*_*ê*_*i*_ to discuss the eigenvalue along the direction of its associated eigenvector in the lattice reference frame, i.e., ***V***_*xx*_ = *V*_*xx*_*ê*_1_, ***V***_*yy*_ = *V*_*yy*_*ê*_2_, ***V***_*zz*_ = *V*_*zz*_*ê*_3_. Similarly, we label the eigenvalues of the instantaneous snapshots from the MD trajectory as *V*_*ii*_*′*, and these are associated with normalized eigenvectors in the lattice frame *ê*_*i*_*′* so that we can define vectors ***V***_*ii*_*′* = *V*_*ii*_*′ê*_*i*_*′*. We also note that the signs of the eigenvectors *ê*_*i*_ and *ê*_*i*_*′* are arbitrary. In order to maintain consistency in presenting our results, we choose the sign of the eigenvectors such that *ê*_*i*_*′*·*ê*_*i*_ > 0, with *ê*_*i*_ being a fixed value, as it comes from the static calculation.

Using this notation, we investigate how the dynamical EFGs ***V***_*zz*_*′* change relative to the static ***V***_*zz*_ throughout the MD simulation. We considered variation in three quantities shown in Figure 1(b): the angle between the directions of the dynamic ***V***_*zz*_*′* (or *ê*_3_*′*) and static ***V***_*zz*_ (or *ê*_3_) denoted by θ_3_, the variation in the magnitude of projection of ***V***_*zz*_*′* onto *ê*_3_ denoted by *V*_proj_, and the angle θ_12_ of *ê*_3_*′* projected onto the *ê*_1_–*ê*_2_ plane. These values are calculated in the following way,
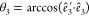
6
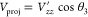
7
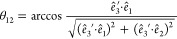
8

## 3 Results and Discussion

The EFG parameters obtained from the static and DFT-MD calculations are compared to experimental results from refs. (78−82) in Table 1. We see that the magnitudes of the calculated static EFG eigenvalues are overestimated in all cases, while the DFT-MD scheme gives values that are closer to the experimental values.

**Figure 2:**
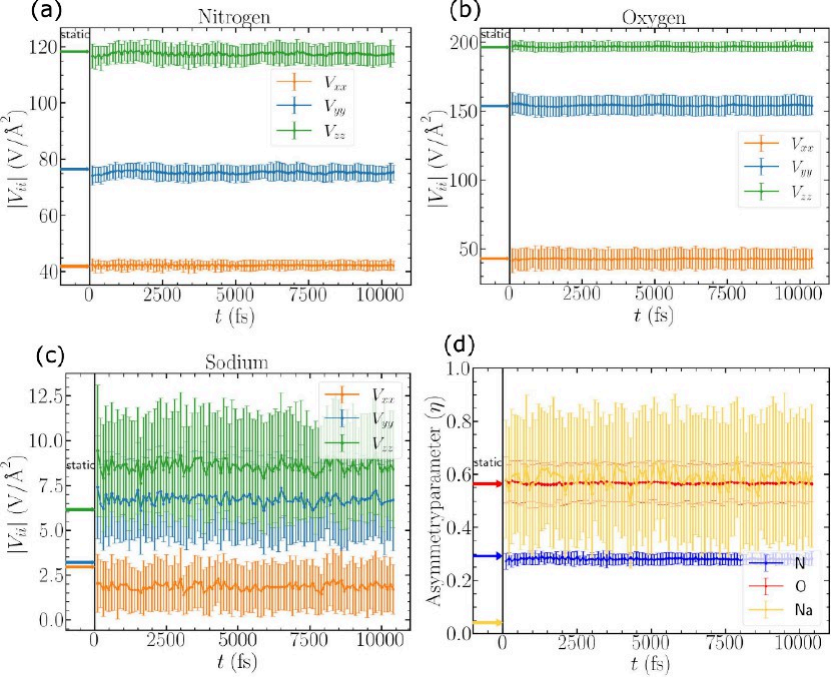
Variation in EFG parameters centered around (a) nitrogen, (b) oxygen, and (c) sodium atoms in NaNO_2_ and (d) variation in asymmetry parameter (η) over the DFT-MD steps for each atom types. The corresponding static values in each case are shown on the left by arrows of the correspondingly matching colors. We provide a more detailed analysis of the fluctuations of *V*_*zz*_*′* in sodium in Sec. 3.4 and Figure 9(b).

We next discuss the DFT-MD results, first investigating the change in EFG parameters in each of N, O, and Na atoms over the MD trajectory. In Figure 2(a-c) we plot the mean values of |*V*_*ii*_*′*| for nitrogen, oxygen, and sodium throughout the MD simulation. The mean is calculated over all atoms of that type in the cell at a given time step. Note that time needed to equilibrate has already been excluded in this plot, so that *t* = 0 can be thought of as already being in thermal equilibrium. The error bars in Figure 2 represent the standard deviation on the mean values of |*V*_*ii*_*′*|, where again the standard deviation is with respect to all atoms of a particular type at a given time step. The static values are shown as arrows to the left of *t* = 0 for comparison. In Figure 2(d) we show mean and standard deviations of η for each atom type, with static values shown as arrows to the left of *t* = 0.

Figure 2(a) and Figure 2(b) show smooth fluctuations in |*V*_*ii*_*′*| for nitrogen and oxygen, respectively. Both of these atoms have similar trends with η also. However, in Na, we see larger fluctuations in |*V*_*ii*_*′*| (Figure 2(c)). We return to a more detailed discussion of the fluctuations of *V*_*zz*_*′* in sodium in Sec. 3.4. The fluctuation in the asymmetry parameter is also larger for Na (Figure 2(d)) due to a swapping between ordering of *V*_*xx*_ and *V*_*yy*_ that leads to larger asymmetry. In addition, the static η for Na, shown by the yellow arrow in Figure 2(d) does not correspond to the mean dynamic η. This opens questions regarding the limitations of static calculations that we discuss in detail in Sec. 3.3.

In the following sections, we investigate the dynamical motion of the EFGs of nitrogen, oxygen, and sodium in greater detail, with visualizations of the dependence of the dynamic EFGs on θ_3_ and θ_12_, as well as how this corresponds to a real space picture of how ***V***_*zz*_*′* is distributed spatially for each atom type. Together, this information provides a wealth of detailed microscopic information that helps to understand how the dynamic motion of atoms leads to averaged macroscopic values.

In order to connect with experiments, it is important to consider the projection of the dynamic EFGs onto the static values. This is because experiments measure NMR or NQR frequencies on the order of kHz to MHz, whereas the atomic vibrations in a solid are THz. Because of this, experiments will measure only the mean values of *C*_*Q*_ or *V*_*zz*_ along a particular direction in the lattice, and therefore an appropriate quantity to connect with experiments is *V*_proj_. Of course, this assumes that the static ***V***_*zz*_ direction (*ê*_3_) is appropriate for calculating *V*_proj_. While in principle it is possible that the static calculation would lead to an *ê*_3_ that is oriented along a different direction than the mean of the dynamic *ê*_3_*′* values, this is not the case for nitrogen or oxygen in NaNO_2_, as we show in the following sections.

### 3.1 ^14^N NMR/NQR in NaNO_2_

**Figure 3:**
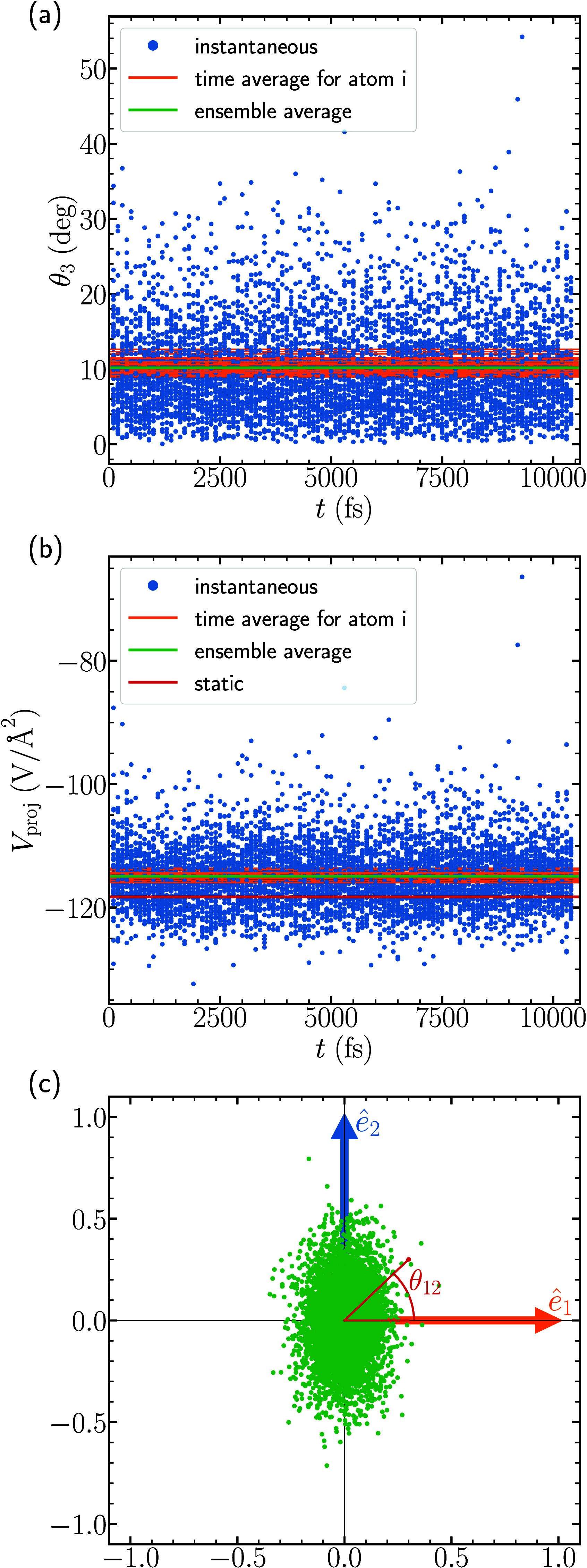
Nitrogen EFGs from the MD trajectory. Time dependence of (a) θ_3_, (b) *V*_proj_, and (c) the distribution of *ê*_3_*′* projected onto the *ê*_1_–*ê*_2_ plane are shown. θ_12_ can be seen in panel (c). Dots in all panels are instantaneous values from the MD trajectory. In panels (a) and (b) the orange and green horizontal lines are time-averaged values over each atom and the full set of atoms, respectively. The static *V*_*zz*_ is shown in panel (b) in red.

^14^N has nuclear spin *I* = 1, quadrupole moment *Q* = 20.44 mb,^83^ and is one of the most frequently studied elements in NMR/NQR. To study the influence of the dynamic motion of atoms on the EFG parameters of nitrogen, we studied the variation of angle θ_3_ made by the *z*-component of the dynamic EFG (***V***_*zz*_*′*) with the static EFG (***V***_*zz*_) as shown in Figure 3(a). The instantaneous change is shown by dots in blue, the time average for each of the *N* atoms over the DFT-MD steps is shown by orange horizontal lines, and the average of these over all atoms, which we denote as the ensemble average, is shown by the green horizontal line. We see that the mean variation of θ_3_ for all *N* atoms during all steps is around 10 degrees. In addition, we note that the mean values and associated standard deviations of *ê*_3_*′*·*ê*_1_ and *ê*_3_*′*·*ê*_2_ for nitrogen for all atoms throughout the MD trajectory are −0.0004 ± 0.091 and −0.001 ± 0.182, respectively. This indicates that the mean direction of *ê*_3_*′* is in nearly exact alignment with the static *ê*_3_, which as discussed previously is necessary for the values θ_3_ and θ_12_ to be meaningful.

We show the projected value of ***V***_*zz*_*′* onto ***V***_*zz*_ (*V*_proj_) in Figure 3(b). The result shows that *V*_proj_ = −115.2 V/Å^2^ is closer to the experimental (absolute) value of 111 V/Å^2^^78^ than the static value we calculate, *V*_*zz*_ = −118.261 V/Å^2^. Note that experiments usually cannot detect the sign of *V*_*zz*_, which is one of the benefits of our calculations. In this case, our static value is in line with the DFT (PBE) range of |*V*_*zz*_| = 117 to 120 V/Å^2^ calculated in ref. (62) for different PAW potentials. This corresponds to a *C*_*Q*_ that moves from the static value of 5.845 MHz to a mean dynamic value of 5.684 MHz, which is closer to the experimentally measured value of 5.5 MHz.^78^ The better agreement in *V*_proj_ is due to the fact that it is more closely related to what is measured in experiments, as mentioned previously.

In addition to the angle θ_3_, we investigate θ_12_ in Figure 3(c). θ_12_ quantifies the angular distribution of *ê*_3_*′* in the *ê*_1_–*ê*_2_ plane, which is perpendicular to the static *ê*_3_. This pattern shows that the majority of ***V***_*zz*_*′* in nitrogen over the whole DFT-MD simulation has a preferential variation along the *ê*_2_ direction, which for nitrogen corresponds to the **a** axis in Figure 1(a). The variation in the *ê*_1_ (**c** axis in Figure 1(a)) direction is less favorable.

**Figure 4:**
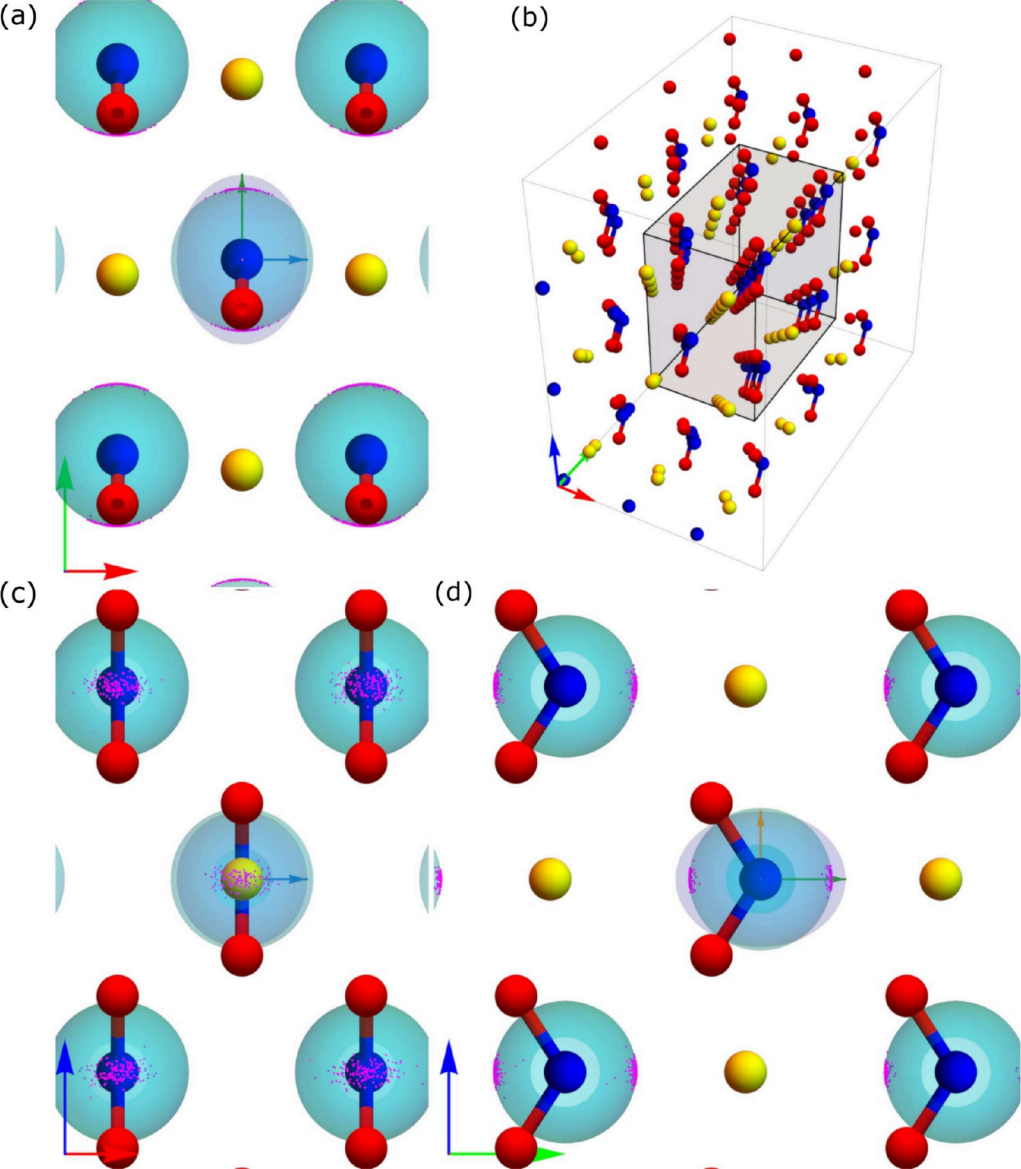
Distribution of *ê*_3_*′* (magenta dots) for single nitrogen atoms over the full DFT-MD simulation. Arrows on the bottom left of each panel correspond to the **a** (red), **b** (green), and **c** (blue), lattice vectors (same as in Figure 1(a)), while the arrows in the central atom in panels a, c, and d, show the eigenvectors of the static calculation where green, blue, and orange, correspond to *ê*_3_, *ê*_2_, and *ê*_1_, respectively (same color scheme as in Figure 2). The central ellipsoid represents a constant electrostatic potential surface at the nucleus, as is discussed in Sec. 3.4.

In Figure 4, we show how the ***V***_*zz*_*′* in nitrogen are distributed in real space throughout the DFT-MD trajectory. Figure 4(b) shows the 3-dimensional NaNO_2_ supercell with lattice vectors along the **a**, **b**, and **c** directions shown by red, green, and blue arrows. The images in Figure 4(a), Figure 4(c), and Figure 4(d) show the distributions of ***V***_*zz*_*′* (in magenta dots) as seen from three different directions, a top view (the **a**-**b** plane), a front view (the **a**-**c** plane), and a side view (the **b**-**c** plane) of the lattice, respectively. The lattice vectors for each case are shown in the bottom-left corner and the axes directions shown in the central part of each individual image are the directions of the static eigenvectors: *ê*_1_, denoted by an orange arrow, *ê*_2_, denoted by a blue arrow, and *ê*_3_, denoted by a green arrow. Note that the colors of the eigenvectors here correspond to the colors of *V*_*xx*_, *V*_*yy*_, and *V*_*zz*_ in Figure 2. We show the distribution of *ê*_3_*′* on the surface of unit spheres in each of the nitrogen positions of the static lattice. Note that we show both the negative and positive eigenvectors for each point as they give the same angle in our definition. The eigenvector, *ê*_3_, of the *V*_*zz*_ eigenvalue, points in the **b**-direction of the crystal lattice, from N toward Na. As we previously noted, the preferred direction for variations in the *ê*_1_–*ê*_2_ plane is *ê*_2_, that is, the **a** lattice direction. This direction corresponds to variations out of the plane of the NO_2_ molecule. The ellipsoid on the center atom represent a constant electrostatic potential surface at the nucleus, and is discussed in Sec. 3.4.

### 3.2 ^17^O NMR/NQR in NaNO_2_

We now discuss the EFG parameters of oxygen in NaNO_2_. Although ^16^O is most abundant in nature (99.76%), it has nuclear spin *I* = 0, so it cannot be detected via NMR nor NQR. We therefore focus our attention on ^17^O (abundance 0.04%), which has nuclear spin *I* = 5/2 and a nuclear quadrupole moment *Q* = −25.58 mb. ^17^O is therefore SSNMR/NQR active so that NaNO_2_ samples synthesized with ^17^O can be used for detection purposes.^82^ Note that in our DFT-MD studies, we keep the nuclear mass of oxygen fixed at the standard value 16 amu, rather than using the ^17^O value of 17 amu. The slightly lower nuclear mass would only slightly effect the phonon frequencies and should not be expected to lead to substantial quantitative changes in the results.

The static *V*_*zz*_ we calculate for ^17^O is −196.267 V/Å^2^ (Table 1). Similar to the case of ^14^N, the magnitude of *V*_*zz*_ in ^17^O is larger than the experimental value of 196 V/Å^2^.^82^Figure 2(b) and Figure 2(d) show the variation of the magnitudes of |*V*_*ii*_*′*| and η, respectively, over the MD trajectory. Motivated by the utility of *V*_proj_ as a metric for comparing to experiments for nitrogen, we now perform a similar analysis for oxygen. As for the case of nitrogen, one concern in evaluating θ_3_, *V*_proj_, and θ_12_ is whether *ê*_3_ in static calculations is a “good” axis to use for projections. Here again, we find that it is a good axis, since mean values and associated standard deviations of *ê*_3_*′*·*ê*_1_ and *ê*_3_*′*·*ê*_2_ for all atoms throughout the MD trajectory are 0.001 ± 0.169 and 0.000 ± 0.097, respectively. This indicates that the mean direction of *ê*_3_*′* is in nearly exact alignment with the static *ê*_3_.

**Figure 5:**
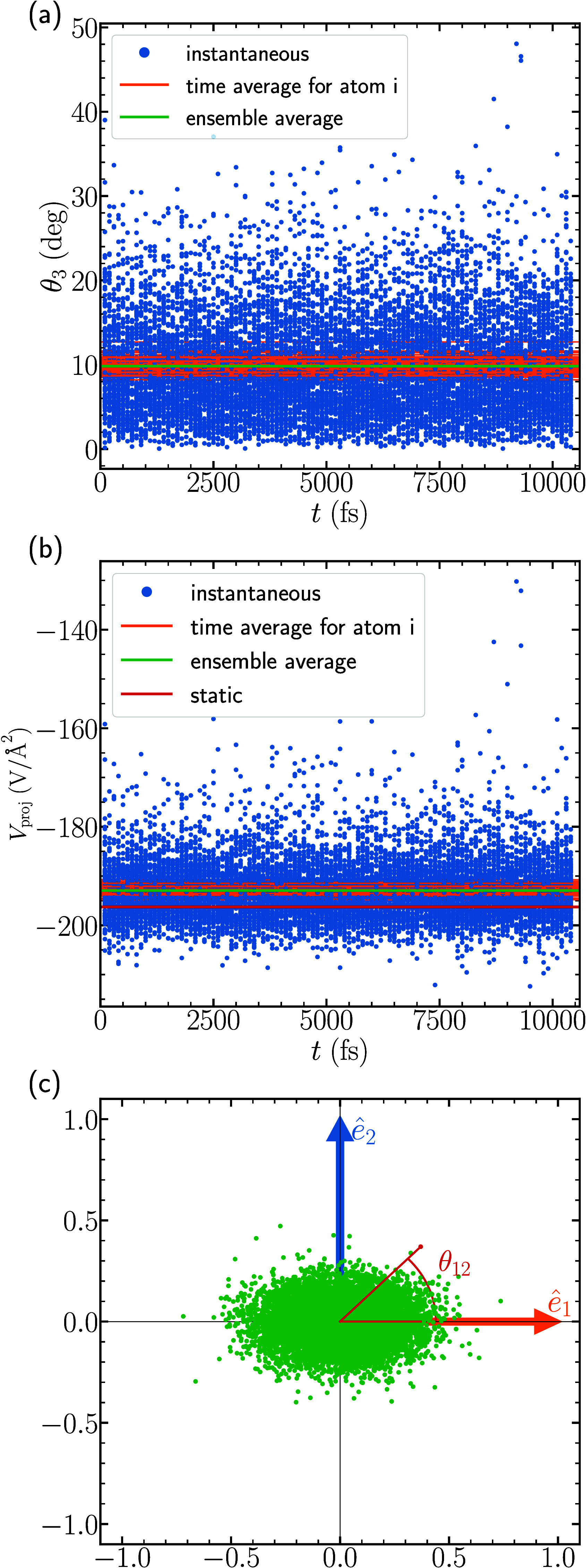
Oxygen EFGs from the MD trajectory. Time dependence of (a) θ_3_, (b) *V*_proj_, and (c) the distribution of *ê*_3_*′* projected onto the *ê*_1_–*ê*_2_ plane are shown. θ_12_ can be seen in panel (c). Dots in all panels are instantaneous values from the MD trajectory. In panels (a) and (b) the orange and green horizontal lines are time-averaged values over each atom and the full set of atoms, respectively. The static *V*_*zz*_ is shown in panel (b) in red.

Figure 5(a) shows the variation of angle θ_3_, Figure 5(b) shows the variation in *V*_proj_, and Figure 5(c) shows the projection of *ê*_3_*′* onto the *ê*_1_–*ê*_2_ plane, with associated angle θ_12_. We calculate an ensemble averaged value *V*_proj_ = −193 V/Å^2^, shown by a green horizontal line in Figure 5(b). The *C*_*Q*_ calculated with the static value *V*_*zz*_ is 12.14 MHz, and that associated with *V*_proj_ is 11.94 MHz, closer to the experimentally measured value of 11.05 MHz.^82^ We also point out that previous static DFT calculations reported *C*_*Q*_ = 12.14 MHz,^82^ the same as the static value we calculate. In Figure 5(c) we see that the projection of *ê*_3_*′* onto the *ê*_1_–*ê*_2_ axis shows preferential alignment along the *ê*_1_ axis. In this case, *ê*_1_ is aligned with the **a** lattice constant, so that the long axis of the ellipse for both nitrogen (Figure 3(c)) and oxygen (Figure 5(c)) is oriented along the **a** axis, even though their individual *ê*_*i*_ frames are oriented differently. The preferential alignment of *ê*_3_*′* for both nitrogen and oxygen along the **a** lattice constant may be related to the amplitude of the vibrational modes of the NO_2_^–^ molecule along that direction, though a detailed study of the correlation between atomic motion and *ê*_3_*′* here is beyond the scope of the present work.

**Figure 6:**
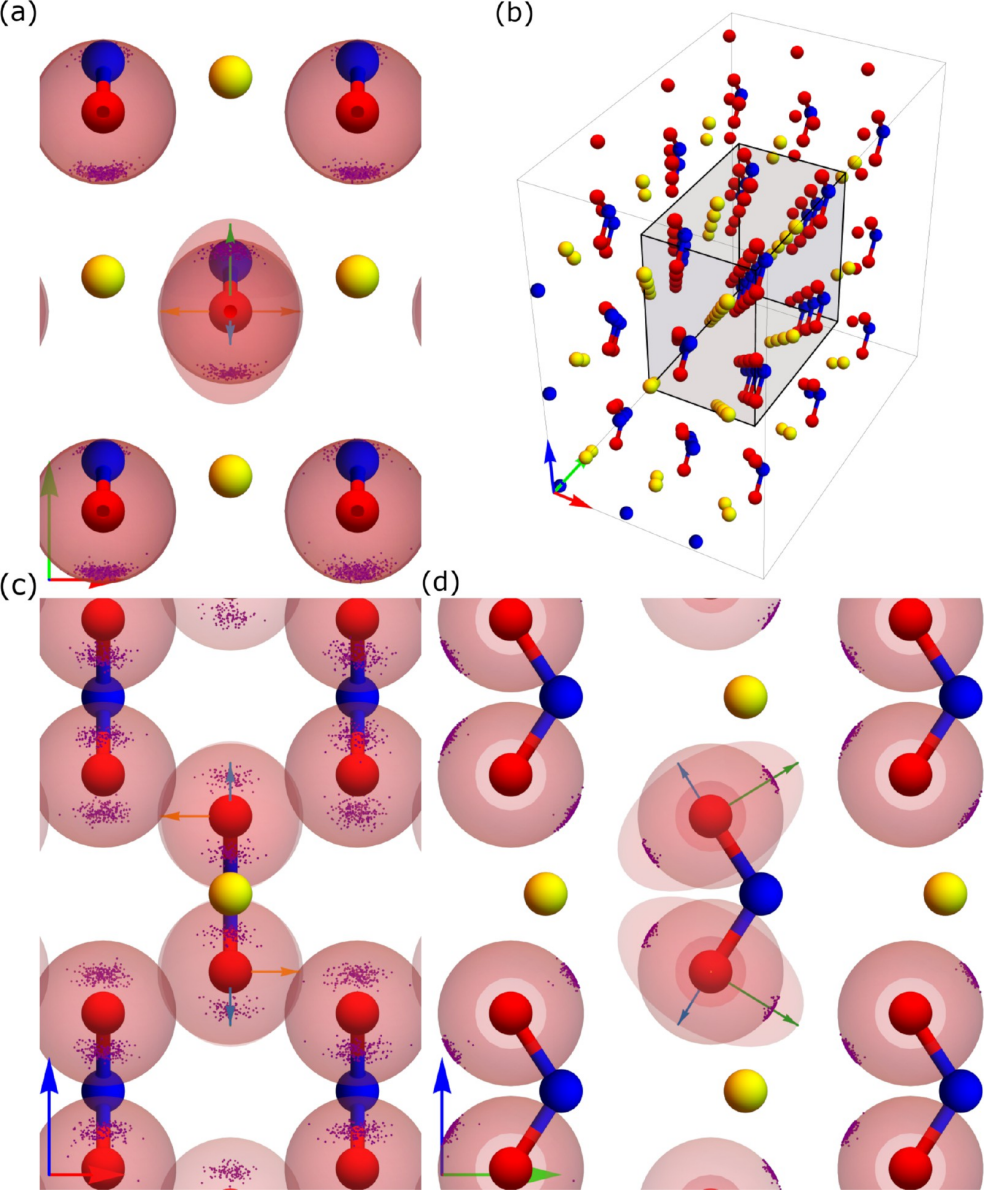
Distribution of *ê*_3_*′* (purple dots) for single oxygen atoms over the full DFT-MD simulation. Arrows on the bottom left of each panel correspond to the **a** (red), **b** (green), and **c** (blue), lattice vectors (same as in Figure 1(a)), while the arrows in the central atom in panels a, c, and d, show the eigenvectors of the static calculation where green, blue, and orange, correspond to *ê*_3_, *ê*_2_, and *ê*_1_, respectively (same color scheme as in Figure 2). The central ellipsoid represents a constant electrostatic potential surface at the nucleus, as is discussed in Sec. 3.4.

In Figure 6 we show the three-dimensional representation of eigenvectors ***V***_*zz*_*′* for oxygen atoms in a central cell of the supercell used for DFT-MD. The purple dots show ±***V***_*zz*_*′* for all snapshots, with each vector centered at the oxygen positions in the static lattice for easier visualization. Figures 6(a), 6(c), and 6(d) show that the *ê*_3_*′* closely align with the *ê*_3_ direction of the static lattice and that, similarly to the case of nitrogen, the largest variation in the *ê*_1_–*ê*_2_ plane is along the **a** lattice constant, that is, out of the plane of the NO_2_^–^ molecule. As mentioned previously, this variation is also seen in Figure 5(c), since in this case *ê*_1_ lies along the **a** lattice constant. The ellipsoids on the central oxygen atoms represent constant electrostatic potential surfaces at the nucleus. We discuss these equipotential surfaces in greater detail in Sec. 3.4.

### 3.3 ^23^Na NMR/NQR in NaNO_2_

Sodium (^23^Na) in NaNO_2_ has strong ionic character. In our static calculations, the angle between the two N–O bonds in NO_2_ is 114.3°, which is in closer agreement with the free nitrite anion (NO_2_^–^) angle of 115° than with the neutral NO_2_ angle of 134°, and it is clear that the negative charge on NO_2_^–^ is from the extra electron in sodium. Sodium has nuclear spin *I* = 3/2 and nuclear quadrupole moment *Q* = 104 mb. In Figure 2(c-d), we see that ^23^Na exhibits large fluctuations in the magnitudes of EFGs |*V*_*ii*_*′*| and η, as compared to nitrogen and oxygen. The reason for this is rather complicated, as we explain below. However, we first note that this finding is also in agreement with other studies^62,84^ that report different values for the quadrupolar coupling constant *C*_*Q*_ and that also mention that the results vary greatly according to the choice of basis sets. A careful analysis of how atomic motion effects the EFGs of ^23^Na gives us a much deeper understanding for what is happening in this case.

**Figure 7:**
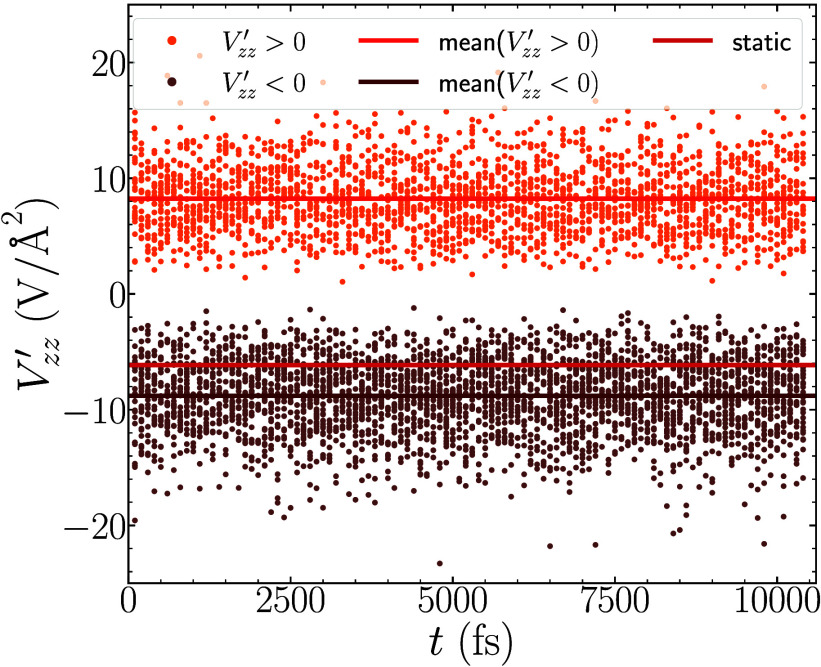
Dynamic values of *V*_*zz*_*′* for sodium. We find that *V*_*zz*_*′* switches from both positive (total 2,289 orange dots) and negative (total 3,327 brown dots) sign. This indicates also that the negative sign occurs roughly 50% more often in our DFT-MD simulations. The mean values for each sign (+8.23 V/Å^2^ and −8.80 V/Å^2^) are plotted as horizontal orange and brown lines respectively, while the static value (−6.15 V/Å^2^) is shown in red for reference.

For nitrogen and oxygen, we plotted in Figures 3 and 5 the dynamic values of θ_3_ and *V*_proj_. This was made possible by the fact that the sign of *V*_*zz*_*′* remained the same throughout the simulation and that the angle θ_3_ remained quite small on average, around 10° for both. In sodium, we find that the sign of *V*_*zz*_*′* switches frequently, as shown in Figure 7. As we discuss in Sec. 3.4, the changing sign is related to a change in the potential surface throughout the MD trajectory that leads to a dynamic *ê*_3_*′* that changes direction substantially throughout the course of the simulation. It is therefore not possible to define a single “good” *ê*_3_ axis to use for projections, and therefore we do not consider the values θ_3_, *V*_proj_, and θ_12_ for sodium. Instead, we investigate only the mean values for the two signs of *V*_*zz*_*′* separately. The mean values are shown in Figure 7 as dark orange and dark brown horizontal lines, which have values +8.23 V/Å^2^ and −8.80 V/Å^2^, respectively. In addition we point out that the number of occurences of the positive and negative signs in the DFT-MD simulation are 2,289 and 3,327, respectively. This indicates that the negative sign occurs about 50% more frequently than the positive sign.

The swapping sign of *V*_*zz*_*′* requires careful consideration when using DFT to calculate a value of *V*_*zz*_ or equivalently *C*_*Q*_ that can be compared with experiments. Usually experiments measure only the magnitude of *C*_*Q*_, which raises questions about what type of MD ensemble average we should use for comparison. The naive analysis of sodium in Figure 2(c-d) ignores the sign change, and taking statistical averages of the magnitudes may not be the relevant quantity to compare with experiments. Another option is to take statistical averages of the signed values. Doing so leads to a value *V*_*zz*_*′* = −4.1 V/Å^2^ that is close to the experimental result |*V*_*zz*_| = 4.37 V/Å^2^ in refs. (80 and 81). However, it is not clear that this is the proper statistical average to use for comparisons with experiments. Further investigation on this issue is necessary, as it requires experimental details (e.g., measurement direction, whether single crystal vs polycrystalline samples were used, etc.) in order to properly model these experiments with our computational approach. We do not attempt to address these questions in the present work, but leave it as an open question for future work.

**Figure 8:**
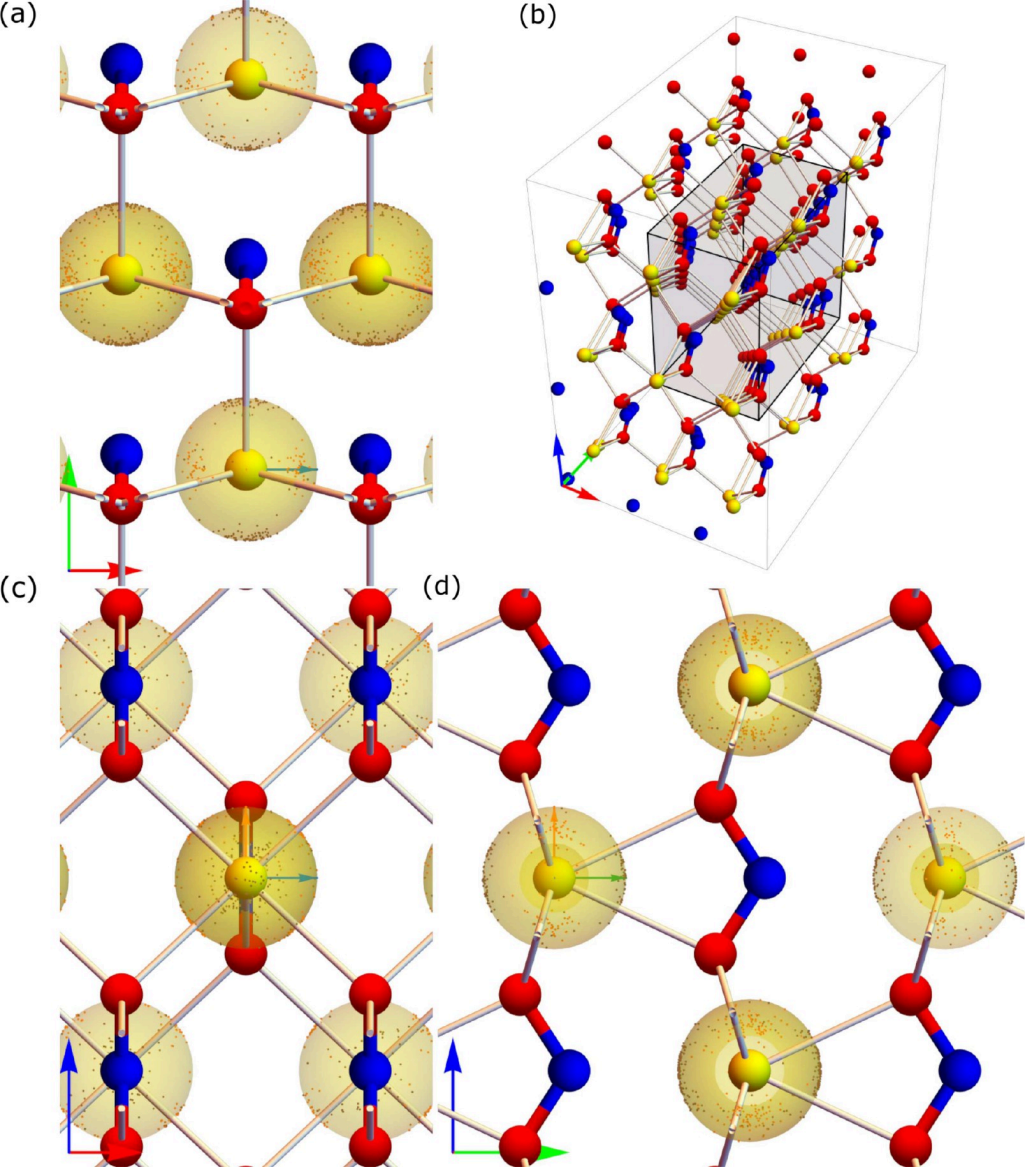
Distribution of *ê*_3_*′* (brown and orange dots) for single sodium atoms over the full DFT-MD simulation. In this case, brown dots correspond to *V*_*zz*_*′* < 0 and orange dots correspond to *V*_*zz*_ > 0. Arrows on the bottom left of each panel correspond to the **a** (red), **b** (green), and **c** (blue), lattice vectors (same as in Figure 1(a)), while the arrows in the central atom in panels a, c, and d, show the eigenvectors of the static calculation where green, blue, and orange, correspond to *ê*_3_, *ê*_2_, and *ê*_1_, respectively (same color scheme as in Figure 2). The central ellipsoid represents a constant electrostatic potential surface at the nucleus, as is discussed in Sec. 3.4.

We can also extend the three-dimensional visualizations for nitrogen (Figure 4) and oxygen (Figure 6) to sodium, which we shown in Figure 8. Here we color the ***V***_*zz*_*′* dots differently depending on whether the associated eigenvalue *V*_*zz*_*′* is negative (brown) or positive (orange). This is a completely different scenario in comparison to the N and O cases presented above and we clearly see that there is not a single unique *ê*_3_ direction that we can use to project *ê*_3_*′* onto. Also, the distribution of *V*_*zz*_*′* eigenvector is more dispersed in comparison to N and O. We discuss this unique behavior of Na in more detail in Sec. 3.4.

### 3.4 Interpreting EFGs in Terms of Equipotential Surfaces

In order to better understand the results for sodium, we make use of a toy model for the electrostatic potential of a nucleus in a nonhomogeneous electron density environment. An electrostatic potential close to a nucleus can be approximated to have a harmonic form, with the environment as a small perturbation on a spherically symmetric potential. Our toy model potential takes the following form,
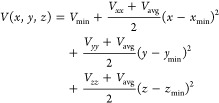
9

Inserting this potential in the definition of *V*_*ij*_, eq 3, we see that *V*_*ii*_ corresponds to the EFG parameters we study in this article. We estimate the value of the curvature of the spherically symmetric part of the potential, *V*_avg_, from two simplified spherically symmetric electron densities, one with Gaussian radial shape containing *q* electrons and one containing only two 1*s* electrons (as all PAW potentials we use in this study assume). In both cases *V*_avg_ is several order of magnitudes larger than the values of *V*_*ii*_ we obtain in NaNO_2_. Instead of using a true, very large, value for *V*_avg_ that would make visualizations difficult, we use a much smaller value that make the deviations from spherical symmetry easier to see in our visualizations. We use *V*_avg_ = 400 V/Å^2^ for all three different atom types in Figures 4, 6, and 8.

Eq 9 suggests that the equipotential surfaces will be elllipsoids with semiaxes given by
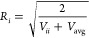
10We show these ellipsoids in Figures 4, 6, and 8, around one (two for O) central atom, normalized so that the unit sphere represents the spherically symmetric average potential. While both N and O have clear asymmetries, we see that Na is very nearly spherical. The very small asymmetry of the Na electrostatic potential at the nucleus is one of the reasons we obtain both positive and negative *V*_*zz*_*′* values.

Since the EFGs satisfy |*V*_*zz*_| ≥ |*V*_*yy*_| ≥ |*V*_*xx*_| and *V*_*zz*_ + *V*_*yy*_ + *V*_*xx*_ = 0, we have two cases to consider,
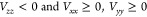
11
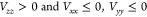
12The condition that the EFG tensor is traceless (see eq 3 and immediately above), reduces the three independent curvatures of the electrostatic potential, (*V*_avg_ + *V*_*ii*_), to only two independent experimentally measurable components, *V*_*zz*_ (or *C*_*Q*_) and η. What is given up with this condition is the magnitude of the spherically symmetric component of the potential, *V*_avg_, which does not couple to the quadrupole moment of the nucleus.

If *V*_*zz*_ < 0, eq 10 suggests that *V*_avg_ + *V*_*zz*_ is the smallest curvature and therefore has the largest axis, *R*_*z*_, in the ellipsoidal equipotential surface. This means that the asymmetry in the potential gives a smaller gradient than average in the *z* (or *ê*_3_) direction. The smallest axis is *R*_*y*_ in the *y* (or *ê*_2_) direction and the asymmetry gives larger gradients than average in both the *x* and *y* directions.

For *V*_*zz*_ > 0, *V*_avg_ + *V*_*zz*_ is the largest curvature and gives that the smallest axis is *R*_*z*_. This means that the asymmetry in the potential gives a larger gradient than average in the *z*- (or *ê*_3_) direction and smaller gradients in both *x* and *y* directions. So, while *V*_*zz*_ by definition is associated with the largest *deviation* from spherical symmetry, the sign of *V*_*zz*_ (or *V*_*ii*_ in general) is associated with this deviation tending toward smaller (−) or larger (+) gradients than the average in the *ê*_*i*_ direction.

In Figure 4 we see that the N ellipsoid has its longest axis along the *ê*_3_ direction (**b**-direction of the lattice) and the shortest axis along the *ê*_2_ direction, just as the static negative value of *V*_*zz*_ tells us. This means that we have a smaller gradient of the electrostatic potential than average for the N nucleus toward the Na atoms. This could also indicate that this would be the preferred direction of motion of the N atom in the MD run, but we will explore this in a more detailed study in the future. The direction with largest gradient is the *ê*_2_ direction, that is, the **a** direction of the lattice. However, in the *ê*_1_–*ê*_2_ plane the preferred direction of motion would then be in the *ê*_1_ direction, since the gradient along *ê*_1_ is smaller than along *ê*_2_. This is in contrast to Figure 3(c), which instead indicates that the motion is likely orthogonal to the plane of the bonds in the NO_2_ molecule, i.e., the *ê*_2_ (or **a**) direction. This is one of the motivations for a follow up study to investigate the correlations between atomic movement and changes in the EFG eigenvectors and eigenvalues. However, this is beyond the scope of the present work.

In Figure 6 we see that each of the two ellipsoids around the oxygen atoms have their longest axes along *ê*_3_ and the shortest axis along *ê*_2_. This is again due to the negative static *V*_*zz*_, as seen for nitrogen. This may indicate that the easiest motion of oxygen is in the **b**–**c** plane orthogonal to the N–O bonds (green arrow in Figure 6(d)). We do not attempt to distinguish whether this is a synchronized motion with the N so that it is the molecule that moves as a unit or if N and O motion are separate or perhaps that this is a sign of a particular molecular vibrational mode. In the *ê*_1_–*ê*_2_ plane (the plane including the N–O bond but orthogonal to the **b**–**c** plane) we see in Figure 5(c) that the projected distribution indicates easier movement in the *ê*_1_ direction.

**Figure 9:**
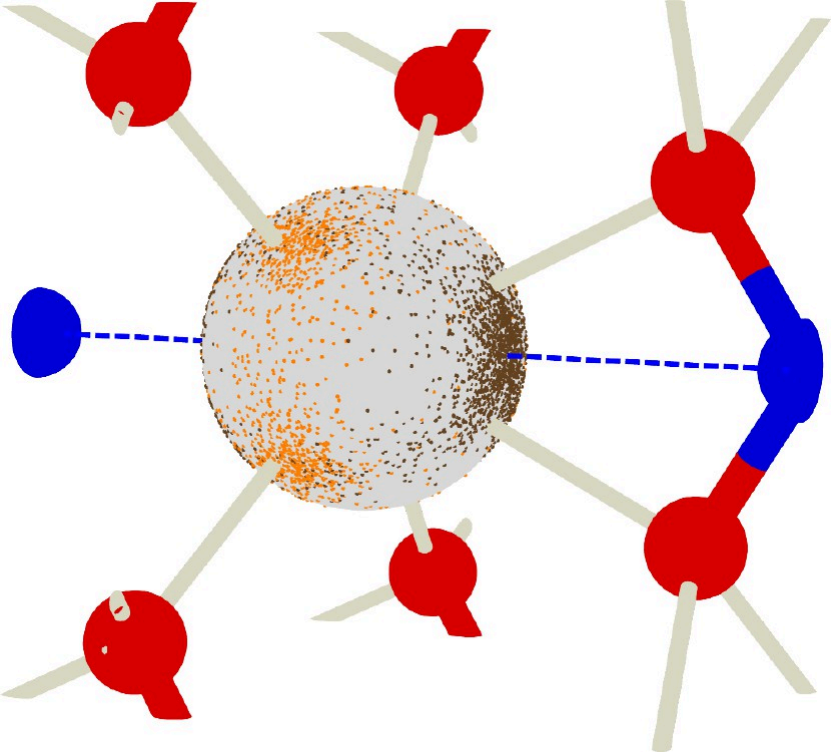
All *ê*_3_*′* eigenvector directions for the Na *V*_*zz*_*′* EFG parameter projected onto the same unit sphere. Positive *V*_*zz*_*′* eigenvector directions are in orange and negative *V*_*zz*_*′* eigenvector directions are in brown.

In Figure 8, similar to the case for nitrogen and oxygen, we show the distribution of *ê*_3_*′* eigenvectors over the DFT-MD steps on the surface of unit spheres of each sodium ion corresponding to the static lattice. The lattice vector notations and eigenvector notations along each component of static *V*_*ii*_ are similar to what we used previously for nitrogen and oxygen. However, contrary to the N and O cases, where *V*_*zz*_*′* is always negative, we here distinguish between positive (orange) and negative (brown) *V*_*zz*_*′* values. In Figure 9 we show the 3,327 *ê*_3_*′* eigenvector directions for the sodium atoms having negative eigenvalues, shown by brown dots, and the 2,289 *ê*_3_*′* eigenvector directions for the sodium atoms corresponding to positive eigenvalues, shown by orange dots. The total number of *ê*_3_*′* eigenvector directions is 5,616, corresponding to 54 Na atoms in each of 104 uncorrelated snapshots of DFT-MD steps. We see from Figure 9 that positive eigenvalue (*V*_*zz*_^*′*^ > 0) has eigenvector directions around the static *ê*_1_–*ê*_2_ plane, in the direction of the closest oxygen atoms. This indicates that eigenvector directions corresponding to positive *V*_*zz*_*′* point in the **a**–**c** plane and give the smallest gradient in the **b**-direction. Similarly, negative *V*_*zz*_*′* has eigenvectors pointing in the **b**-direction of the crystal lattice shown by brown dots in Figure 8, which also gives the smallest gradient in the **b**-direction. So, while the smallest gradient is always in the direction from Na to the closest N, the largest *deviation* from spherical symmetry does not always occur in this direction. The large variations of EFG parameters for Na is thus an artifact of their definitions and the fact that the full ionization of Na to Na^+^ in NaNO_2_ results in an almost completely spherical electrostatic potential at the Na nucleus. It is, however, very interesting that we can see the direction to neighboring O atoms in the distribution of eigenvectors during an MD run. We will explore this in the context of experimental studies in future work.

## 4 Conclusions

In this study, we developed a method to study the effect of atomic dynamics on EFG parameters. Using this method, we systematically studied EFGs of ^14^N, ^17^O, and ^23^Na in NaNO_2_. Due to the very different time scales of NQR or NMR (kHz to MHz) and atomic vibrations (THz), we can use DFT-MD simulations to calculate meaningful statistical averages of EFG eigenvalues and eigenvectors that can be compared to experiments. For ^14^N and ^17^O we find in our calculation that the mean value of the eigenvalue, projected onto the static eigenvector direction, gives a value that is in closer agreement with experiments than the static values themselves. This can be interpreted as the fact that atomic motion causes small fluctuations in the instantaneous eigenvector directions that, when measured on longer time scales, will lead to a reduced *C*_*Q*_ value measured in experiments. The cases of nitrogen and oxygen also showed that the eigenvectors *ê*_3_*′* have a preferred alignment in the plane perpendicular to the static eigenvector *ê*_3_. In the present article, we have not attempted to explain this finding in great detail, though we plan to perform a follow up study in which we explore the correlations between atomic motions and changes in the dynamic *ê*_3_*′* values, which we expect to give insight into how the microscopic motion of individual atoms changes the local equipotential surface and thereby effects the EFG eigenvector directions and eigenvalue magnitudes.

We also found that the EFG eigenvalues and eigenvectors for ^23^Na are much more complicated than for nitrogen and oxygen. The complications arise from a combination of the small magnitudes |*V*_*zz*_| and the fact that its motion in the cell leads to instantaneous values of *V*_*zz*_*′* that switch between positive and negative sign. This can be contrasted to the cases of nitrogen and oxygen that both have *V*_*zz*_*′* < 0 for all snapshots throughout the MD trajectory. By investigating the eigenvectors associated with each sign of *V*_*zz*_*′* in sodium, we find that a clear pattern emerges in which *V*_*zz*_*′* < 0 leads to eigenvectors *ê*_3_*′* aligned with the Na–N nearest neighbor direction, while the case *V*_*zz*_*′* > 0 leads to eigenvectors *ê*_3_*′* aligned along the Na–O nearest neighbor directions. This finding indicates that the microscopic motion of sodium precludes the use or definition of a single “good” eigenvector direction to use for interpreting experimental results. This finding suggests that for sodium in NaNO_2_, and atoms in other materials, a much more thorough and careful understanding and interpretation of experiments is needed in order to connect with theoretical studies.

Again, we point out that in this study, we have not explored the correlations of atomic motions to EFG eigenvalues and eigenvector directions. We expect that such studies are needed to further our understanding of NaNO_2_ and other materials, especially in cases where atomic motion leads to drastic changes in these quantities, as is the case for sodium here. We also anticipate that the methodology we present here may be useful for better understanding materials systems in which static EFG calculations do not align with experiments. This is a particularly strong concern for systems with hydrogen bonded to ^14^N or other quadrupolar nuclei, as in those cases hydrogen is highly mobile and could strongly influence both the instantaneous eigenvalues and eigenvectors of the EFG tensor.
